# Carbohydrate beliefs and practices of ultra-endurance runners in Ireland for gastrointestinal symptom management

**DOI:** 10.3389/fnut.2024.1408101

**Published:** 2024-05-31

**Authors:** Tansy Ryan, Ed Daly, Lisa Ryan

**Affiliations:** Department of Sport, Exercise and Nutrition, School of Science and Computing, Atlantic Technological University, Galway, Ireland

**Keywords:** nutrition, ultra-endurance, gastrointestinal, dietary habits, running

## Abstract

**Purpose:**

This study aimed to investigate the carbohydrate beliefs and practices of ultra-endurance runners in Ireland to gain an understanding of their gastrointestinal symptom (GIS) management strategies.

**Methodology:**

An adapted version of a previously developed and validated questionnaire was distributed online to ultra-endurance runners, recognized as those who completed a single bout of exercise lasting 4 or more hours. The questionnaire asked about carbohydrate awareness and sourcing, and dietary practices, particularly in relation to GIS management.

**Results:**

A total of *n* = 68 individuals completed the adapted questionnaire. Of these, *n* = 1 was excluded due to their reporting of an ulcerative colitis diagnosis. The remaining participants included 46 men and 21 women. Personal previous experience was the main source directing participants’ nutrition practices (*n* = 30), while only 3 participants quoted sourcing information from qualified professionals. Forty-two participants experienced GIS, usually equally around training and competition times. Many participants had not previously implemented any specific dietary or non-dietary strategies to alleviate exercise-induced GIS. Supplementing with nitrates (*n* = 9) and probiotics (*n* = 4) were the most common dietary practices to alleviate GIS, while other practices (*n* = 14) and portion control (*n* = 13) were the most reported non-dietary practices.

**Discussion:**

Similar to previous studies, these findings suggest that GISs are prevalent in the ultra-endurance running community, occurring regardless of whether during training or an event. Similarly, this research highlights the vast range of GISs experienced by this population. However, the absence of both dietary and non-dietary-related practices used for GIS management alludes to a current deficit in the availability of nutrition information specific to this problem. Further research is required to understand the mechanisms behind ultra-endurance-associated GISs and its various management strategies as well as best practices for communicating these to the target audience to reduce individuals’ risks of developing long-term, chronic health complications.

## 1 Introduction

Ultra-endurance running, or ultra-marathon running, has been steadily increasing in popularity throughout recent decades (1). These events are generally categorized either by their distance, which exceeds the traditional 26.2 miles (42.2 km) of a typical marathon or by their duration (2). With regard to the latter, some investigators consider the ultra-endurance activity as exertion that lasts for over 4 h (3). The latter presents extreme challenges, with courses often including extreme altitudes, temperatures, and terrain. Physiologically, the literature suggests that this activity risks chronic maladaptations to a myriad of systems, namely immune, digestive, respiratory, and cardiovascular (4). Ensuring sufficient nutritional planning and intake is a critical component during training and preparation periods for ultra-events, supporting athletes both during and after their activity (5).

Recognized as gastrointestinal symptoms (GISs) resulting from physical activity, studies suggest that exercise-induced gastrointestinal symptoms (Ex-GISs) are experienced by between 30 and 90% of distance runners (6). Factors exacerbating GIS may be extrinsic, such as exercise duration and environmental conditions, or intrinsic, relating to diet intake and feeding tolerances. Many of the gastrointestinal effects associated with exercise progressively worsen as exercise intensity increases. These include issues such as mesenteric blood flow reduction, gastric emptying time, and decreased esophageal peristaltic activity. Collectively, these changes impact the digestive and absorptive processes, which may subsequently impair athletic performance and recovery (7). Among the most commonly reported symptoms experienced are bloating, nausea, regurgitation, and abdominal pain (8). While the vast majority of gastrointestinal issues reported are deemed mild with an absence of long-term health consequences, there remains a potential for serious medical conditions to emerge, namely ischemic bowel disease, hematochezia, and hemorrhagic gastritis (7).

Although there have been significant advances regarding the nutritional requirements for endurance athletes, a plethora of gaps exist in the literature which are specific to those partaking in ultra-endurance exercise (9). Currently, the International Society of Sports Nutrition outlines an array of nutritional recommendations both for training and competing in a single-stage ultra-marathon event. These primarily revolve around increasing one’s energy, carbohydrate, fluid, and electrolyte intake and practicing dietary patterns such as those low in fermentable oligosaccharides, disaccharides, monosaccharides, and polyols (FODMAPs) (10). However, research investigating the degree to which ultra-endurance athletes are aware of, source information on, and use strategies relating to these recommendations is scarce (11, 12). Previous studies using questionnaires to explore such topics suggest that ultra-athletes, particularly amateurs, may not place much importance on their dietary intake specifically for their sport participation and often favor the opinion of other athletes over nutrition experts as their information source (13–15). A 2016 systematic review by Trakman et al. (16) highlighted the lack of high-quality research investigating athletes’ nutrition awareness, advocating the need for a universal, validated sports nutrition knowledge assessment tool. The aim of this study was to investigate the carbohydrate beliefs and intended practices of ultra-endurance runners in Ireland using an adapted electronic version of the validated questionnaire developed by Scrivin et al. (15).

## 2 Methods

This research study utilized an adapted electronic version of a previously developed and validated online questionnaire that investigated endurance athletes’ practices to manage GISs around exercise (15). This study was approved by the research ethics committee at Atlantic Technological University, Galway City (ATU231GC).

### 2.1 Recruitment strategy

An infographic was developed which outlined the eligibility criteria for this research, what it entails, and the primary authors’ contact details for those seeking further information. This infographic was distributed online to various running groups, relevant organizations, and ultramarathon event coordinators throughout Ireland for circulation on their websites and social media platforms, as well as advertisements at upcoming events. In addition to this, individual athletes with an active social media presence sharing their experience of ultra-running were privately sent the infographic. Finally, the primary researcher of this study attended two separate ultra-marathon events (Connemarathon and Kerry 50 K Ultra), where participants were informed about the study.

### 2.2 Participant information

Eligibility criteria consisted of being ≥18 years of age, healthy with no current diagnosed health conditions (except for an irritable bowel syndrome (IBS) diagnosis, which was accepted), and training for or competing in running events that are over 4 h in duration.

### 2.3 Questionnaire

Individuals were provided with a link to a Microsoft Forms page, where they gave their informed consent to participate, and completed the questionnaire. The adapted electronic version of the questionnaire previously developed by Scrivin et al. (15) consisted of 33 questions. Athletes were first asked demographic questions, such as age and biological sex, followed by a series of questions on their level of training. Athletes categorized a variety of foods and beverages as either a main source of, or not a source of, carbohydrates. Athletes’ energy and macronutrient intake at various time points were estimated by presenting them with statements that they could answer on a Likert scale from strongly disagree to strongly agree. Athletes were also asked a range of questions about their nutrition and non-nutrition-related practices, information sourcing, and experiences of GISs.

### 2.4 Statistical analysis

The data were analyzed using IBM SPSS Statistics version 28 (IBM Corporation, New Orchard Road, Armonk, NY 10504-1722, United States). The Shapiro–Wilks test was used to determine that the data were normally distributed. Descriptives are outlined as the number of participants who selected each response and where appropriate, the accompanying valid percentage is given and rounded to the nearest whole number.

## 3 Results

A total of 68 (*n* = 46 men and *n* = 21 women ranging from 25 to 65+ years old) ultra-endurance runners completed the adapted electronic questionnaire, of which 1 was excluded from analysis as they reported a current ulcerative colitis diagnosis. Of the remaining participants, 4 reported having previously been diagnosed with a gastrointestinal-related intolerance, disease, or illness (lactose intolerance, gluten intolerance, IBS, and previous colorectal cancer).

### 3.1 Demographics and event information

Of the 67 participants, 46 were men (69%) and 21 were women (31%) ranging from 25 to 65+ years old. The most frequently selected age category was 45 to 54 years old, which contained 24 participants, while there was only 1 participant in the 66+ years old age category. The most common main event type reported was single-day events, with only 10 (15%) runners reporting multi-day events. The most frequent level of aerobic training reported in hours per week during one’s heaviest training period was 10–20 h, which accounted for 61% of responses (Table 1).

**Table 1:** Table outlining the demographics and background training information of participants.

Topic	Classifications	Response *n* (%)
Biological sex	Male	46 (69%)
	Female	21 (31%)
Age range	18–24	0
	25–34	12 (18%)
	35–44	17 (25%)
	45–54	23 (34%)
	55–66	13 (19%)
	66+	1 (1%)
Event type	Single stage	57 (85%)
	Multi-stage	10 (15%)
Hours spent on aerobic training during their heaviest training week	<10	15 (22%)
	10–20	41 (61%)
	20–30	8 (12%)
	30–50	0
	50+	2 (3%)

### 3.2 Information sourcing

Almost half of the participants (*n* = 30, 46%) reported that the main source or factor that directs their nutrition practices is their own past experiences. Seconding this, the experience and recommendations of other athletes (*n* = 10, 15%) were reported as a significant factor, followed by Internet pages (*n* = 10, 15%). Only *n* = 3 participants (5%) were selected as “qualified professionals” as a key source of nutrition information and guidance throughout their training and event preparation.

### 3.3 Carbohydrate awareness

The results are outlined in Table 2 as the total number of responses for each potential answer and its accompanying percentage in relation to all who responded to that particular question. The majority of participants, when asked to categorize various foods either as a key carbohydrate source or not, answered correctly.

**Table 2:** Table outlining the responses of participants when asked to categorize a variety of foods and beverages as either a common carbohydrate or not a carbohydrate source, as a total number of answers and their accompanying valid percentage.

Food	Carbohydrate source *n* (%)	Not carbohydrate source *n* (%)
Grilled chicken	5 (8%)	62 (95%)
Whole meal bread	65 (97%)	2 (3%)
Rolled oats	64 (96%)	3 (5%)
Baked potato	64 (96%)	3 (5%)
Salmon	1 (2%)	66 (99%)
Pasta	66 (99%)	1 (2%)
Rice	63 (94%)	4 (6%)
Lettuce	13 (19%)	54 (81%)
Sports drink	56 (84%)	11 (16%)
Banana	59 (89%)	7 (11%)
Carbonated drink	46 (69%)	21 (31%)
Wheat biscuits	61 (91%)	6 (9%)
Avocado	18 (27%)	48 (73%)
Broccoli	22 (33%)	44 (67%)
Eggs	9 (14%)	57 (86%)

Responding to this question was not compulsory and as a result, not all participants reported their experience.

### 3.4 Nutrition practices

Participants were asked to consider their energy and macronutrient intakes at four different time points (the day before an event, the last meal before an event, during an event, and after an event) and indicate whether they often intend to eat more, less, or the same amount of a nutrient during each period. The day before an event, most participants increased their energy (*n* = 33, 49%) and carbohydrate (*n* = 39, 58%) intakes, with no reports of a reduction in either, while most kept their protein and fat intake at the same level (*n* = 47, 70% and *n* = 48, 72% respectively). Similarly, for the last meal before an event, the majority of participants increased their carbohydrate intake (*n* = 23, 55%) but had the same or less energy (*n* = 36, 54%) than in other meals. In addition to this, more participants had the same or less protein (*n* = 57, 85%) and fat (*n* = 59, 88%). Continuing this pattern, most participants consumed more energy (*n* = 41, 61%) and carbohydrates (*n* = 35, 52%) and the same or less protein (*n* = 61, 91%) and fat (*n* = 63, 94%) during events. After completing an event, participants still consumed more energy (*n* = 40, 60%) and carbohydrates (*n* = 51, 63%), but also more protein (*n* = 42, 75%). Though the number of those increasing their fat intake (*n* = 21, 31%) was higher than at other time points, most participants’ intake remained the same or less (*n* = 45, 67% collectively).

### 3.5 Managing GIS

The majority of participants did not previously implement any specific restrictive dietary pattern to manage their GISs. Participants were given the option to state if and when they had used each strategy (before, during, or after exercise), whether the strategy was successful or not at reducing their GISs, report the statement as not relevant to them, or they could choose not to answer and move onto the next statement. Table 3 outlines the number of participants who did and did not try the various nutritional approaches, as well as the number of those who considered each approach successful for managing their GISs. The most commonly used dietary strategy was probiotic use, of which 4 (6%) participants had previous experience using before (*n* = 2, 3%), during (*n* = 1, 1%), or after (*n* = 1, 1%) exercise (Table 3). Apart from dietary-related practices, participants were questioned on whether they had implemented any other practices into their lives in order to manage their GIS (Table 4).

**Table 3:** Dietary patterns used by ultra-endurance runners in an attempt to mitigate Ex-GIS, outlining the number of individuals who tried and did not try each strategy, and which of those tried were considered successful.

Dietary pattern	Not tried *n* (%)	Tried and considered successful *n* (%)
Gluten-free	57 (85%)	3 (4%)
Dairy-free	46 (69%)	6 (9%)
Lactose-free	54 (81%)	3 (4%)
Wheat-free	64 (96%)	3 (4%)
Low FODMAP*	62 (93%)	0
Glutamine use	59 (88%)	0
L-citrulline use	64 (96%)	0
Arginine use	64 (96%)	0
Curcumin use	63 (94%)	0
Bovine colostrum use	59 (88%)	1 (1%)
Probiotics use	50 (75%)	4 (6%)
Prebiotics use	59 (88%)	0
Symbiotics use	61 (91%)	0
Antioxidants use	50 (75%)	2 (3%)
Nitrates use	48 (72%)	5 (7%)

Responding to this question was not compulsory and as a result, not all participants reported their experience. *FODMAP = Fermentable, oligo-, di-, and mono- saccharides and polyols.

**Table 4:** Non-dietary patterns used by ultra-endurance runners in an attempt to mitigate Ex-GIS, outlining the number of individuals who tried and did not try each strategy, and which of those tried were considered successful.

Other practice	Not tried *n* (%)	Tried and considered successful *n* (%)
Medicine	47 (70%)	12 (18%)
Relaxation	55 (82%)	7 (10%)
Herbal remedies	61 (91%)	2 (3%)
Acupuncture	64 (96%)	0
Sport psychologist	62 (93%)	2 (3%)
Portion control	50 (74%)	13 (19%)
Liquid-only diet	59 (88%)	3 (4%)
Other	45 (67%)	14 (21%)

Responding to this question was not compulsory and as a result, not all participants reported their experience.

### 3.6 Most common times GISs are experienced

When asked at which time point participants most commonly experienced negative GISs, only 26 (41%) of participants responded, “I do not experience them.” Of those who did report experiencing GISs, the majority (*n* = 16, 25%) recounted experiencing GISs equally around training and competition times, followed by (*n* = 16, 25%) solely around competitions, and (*n* = 6, 9%) only around training times.

Participants were provided with a specific time point (before, during, or after a training session or an event) and a range of GIS which they had the opportunity to report as not experienced, mild, severe, or extreme. Regarding GIS before training, there were no reports of “severe” or “extreme” symptoms. The most reported “mild” GIS is an urge to defecate (*n* = 24, 36%), flatulence (*n* = 17, 25%), loose stools (*n* = 12, 18%), and heartburn (*n* = 10, 15%). During training, the only GIS reported as “extreme” was that of vomiting and nausea, with 1 runner reporting each. Of those reported as “mild,” having a stitch (*n* = 18, 27%), an urge to defecate (*n* = 16, 24%), loose stools (*n* = 16, 24%), and nausea (*n* = 13, 19%) were the most frequently experienced. After training, vomiting (*n* = 1, 2%) was the only GIS reported as “extreme.” Flatulence (*n* = 34, 51%), urge to defecate (*n* = 22, 33%), stomach pain (*n* = 17, 25%), loose stools (*n* = 11, 16%), bloating (*n* = 10, 15%), and diarrhea (*n* = 10, 15%) were most frequently reported as “mild.” This period produced the largest amount of GISs for runners. Before an event, no GIS was reported as “severe” or “extreme,” while the urge to defecate (*n* = 24, 36%), flatulence (*n* = 17, 25%), loose stools (*n* = 12, 18%), and heartburn (*n* = 10, 15%) were most reported as “mild.” During an event, no symptoms were reported as “severe” or “extreme.” Reported as mild most frequently was an urge to defecate (*n* = 18, 27%), diarrhea (*n* = 16, 24%), belching (*n* = 15, 22%), flatulence (*n* = 15, 22%), bloating (*n* = 12, 18%), dizziness (*n* = 11, 16%), and nausea (*n* = 10, 15%). After an event, nausea was reported as “extreme” by 1 participant, and there were no reports of “severe” GISs. Of those reported as mild, bloating (*n* = 22, 33%), urge to defecate (*n* = 16, 24%), nausea (*n* = 16, 24%), loose stools (*n* = 16, 24%), vomiting (*n* = 15, 22%), a stitch (*n* = 15, 22%), diarrhea (*n* = 14, 24%), and left intestinal pain (*n* = 12, 18%) were most common (Table 5).

**Table 5:** Most commonly reported mild and extreme GIS experienced by ultra-endurance runners at various time points (before training, during training, after training, before the event, during the event, and after the event).

Time point	GIS severity	Most reported GIS and frequency, *n* (%)
Before training	Mild	Urge to defecate (*n* = 24, 36%), Flatulence (*n* = 17, 25%), Loose stools (*n* = 12, 18%), Heartburn (*n* = 10, 15%)
During training	Extreme	Vomiting (*n* = 1, 2%), Nausea (*n* = 1, 2%)
	Mild	Stitch (*n* = 18, 27%), Urge to defecate (*n* = 16, 24%), Loose stools (*n* = 16, 24%), Nausea (*n* = 13, 19%)
After training	Extreme	Vomiting (*n* = 1, 2%)
	Mild	Flatulence (*n* = 34, 51%), Urge to defecate (*n* = 22, 33%), Stomach pain (*n* = 17, 25%), Loose stools (*n* = 11, 16%), Bloating (*n* = 10, 15%), Diarrhea (*n* = 10, 15%)
Before event	Mild	Urge to defecate (*n* = 24, 36%), Flatulence (*n* = 17, 25%), Loose stools (*n* = 12, 18%), Heartburn (*n* = 10, 15%)
During event	Mild	Urge to defecate (*n* = 18, 27%), Diarrhea (*n* = 16, 24%), Belching (*n* = 15, 22%), Flatulence (*n* = 15, 22%), Bloating (*n* = 12, 18%), Dizziness (*n* = 11, 16%), Nausea (*n* = 10, 15%)
After event	Extreme	Nausea (*n* = 1, 2%)
	Mild	Bloating (*n* = 22, 33%), Urge to defecate (*n* = 16, 24%), Nausea (*n* = 16, 24%), Loose stools (*n* = 16, 24%), Vomiting (*n* = 15, 22%), Stitch (*n* = 15, 22%), Diarrhea (*n* = 14, 24%), Left intestinal pain (*n* = 12, 18%)

Responding to this question was not compulsory and as a result, not all participants reported their experience.

## 4 Discussion

The purpose of this study was to investigate, using an adapted electronic version of the validated questionnaire developed by Scrivin et al. (15), the carbohydrate beliefs and intended practices of ultra-endurance runners in Ireland.

### 4.1 Nutrition awareness

Approximately half (*n* = 30, 46%) of the participants reported that the main information source that influences the nutrition practices they use is experimenting with their dietary intake themselves, followed by recommendations from other athletes (*n* = 10, 15%) and advice from Internet pages (*n* = 10, 15%). In line with the recent study of Mahoney et al. (16), which found healthcare professionals were the least accessible and regularly used method to inform nutrition practices for recreational ultra-runners, the present study further highlighted this matter, as consultation with a nutrition professional was sparse (*n* = 3, 5%). Comparably, web-based searching for nutrition information is a common practice throughout this cohort, whether from websites directly or through social media platforms. A highly accessible source of information, these platforms are an underutilized resource for such research dissemination. Nutrition professionals are adapting their practices to reflect the global increase in digital technology use. Previous research suggests that it may be beneficial to offer training to support this cohort in further embracing social media (17). The findings of this investigation highlight that this could be of particular relevance to those hoping to disseminate information to ultra-endurance populations. A variety of food and beverages were presented to participants, and they labeled each item as either a carbohydrate source or not a carbohydrate source in their diet. Most of the participants categorized the products correctly, suggesting a generally strong level of awareness of their nutritional intake. Those non-carbohydrate sources more frequently classified incorrectly were broccoli (33% incorrect) and avocado (27% incorrect), while carbohydrate sources and carbonated drinks (31% incorrect) were most regularly incorrectly categorized. These were also often mis-categorized by endurance athletes in previous investigations (18). With previous research suggesting that these athletes oftentimes fail to meet recommendations, prioritizing such dietary advice is of paramount importance for practitioners to effectively support athletic performance (19, 20). This becomes even more relevant considering research observations alluding to an interplay between endurance and ultra-endurance athletes’ carbohydrate intake and their experience of GIS (8).

### 4.2 GIS management

Increasing carbohydrate intake was the most common dietary strategy practiced by the ultra-endurance runners involved in this research prior to, during, and after event participation. This practice has previously been reported in the literature, with the concept of increasing carbohydrate intake generally aligning with sports nutrition recommendations. Increasing carbohydrate intake is an already well-established method for athletes to improve their athletic performance and subsequent recovery (21). However, the exact quantities of carbohydrates consumed were not reported in this research. Further investigation is required to establish specific recommendations, particularly with regard to the interplay between carbohydrate intake and GIS experience (8, 22).

The practice of altering one’s dietary pattern was scarce among participants in this research. Nitrates and probiotic use were the most commonly reported practices but were only attempted by 9 and 4 participants, respectively. Overall, there was an obvious absence in the use of nutrition practices to manage GIS by participants, alluding to a current deficit in the availability of nutrition information specific to GIS management. Though not the focal point of the questionnaire used in this investigation, multiple studies exist that suggest that the low FODMAP diet is a promising method for abating Ex-GIS in this specific population (23, 24). Investigating this relationship in a similar cohort, Scrivin et al. (25) observed that a low FODMAP diet was among the most promising nutrition strategies for abating Ex-GIS, along with dietary fiber reduction, dairy-free diets, and increased carbohydrate intakes. Though this exploratory study focused on endurance, not ultra-endurance, athletes, these findings highlight promising strategies for future investigation in an ultra-endurance cohort. Similarly, in their systematic review of the role of dietary supplements on markers of exercise-associated gut damage and permeability, where changes in symptoms experienced were often an outcome measure of included studies, Chantler et al. (26) outline a myriad of supplements that may alleviate GISs in endurance athletes. It is also important to note that vitamins appear to have a modulating effect on the microbiome and, as a result, may potentially affect GIS, though the research in this area remains sparse (27).

Outside of nutrition strategies, very few participants used other practices to manage their GISs. Other strategies (*n* = 14), portion control (*n* = 13), and taking medicine (*n* = 12) were the most frequently reported strategies. In the aforementioned study by Scrivin et al. (25), the most promising non-dietary strategies for endurance Ex-GIS management observed were the use of medication and practicing relaxation/meditation. With “other” receiving the most responses in this research, further investigation to uncover the specific non-dietary practices of ultra-endurance runners for Ex-GIS management requires more attention. Though research into portion control and Ex-GIS is currently non-existent, there is the possibility that this method was used to control specific food types from being consumed in large boluses in one sitting, a recommendation oftentimes provided to reduce one’s FODMAP intake (28).

GIS was most reported as experienced equally around training and competition times. With regard to which specific symptoms were most prevalent, vomiting and nausea were the only ones recorded as being “extreme.” This occurred during training, after training, and after an event. Apart from this, a myriad of GISs were reported throughout training and racing periods, which were considered “mild” by participants. While comparable research is scarce, that which does exist also suggests a wide range of GIS is experienced during ultra-endurance exercise. Stuempfle and Hoffman (29) highlighted this in their investigation of GI distress during a 161 km ultramarathon. Not only was a range of GIS present among both finishers and non-finishers, but 36% of non-finishers reported GIS as their reason for dropping out of the race. Similar to the present study, nausea was among the most common GIS experiences (60% frequency overall and 91% frequency in non-finishers). Such symptoms have frequently been reported during endurance exercise (7, 8).

## 5 Limitations

The present study is not without limitations. Participants’ answers suggested discomfort with discussing GI issues, as when originally asked if they suffer from such problems, many answered that they did not. However, when reporting specific symptoms, it became apparent that many did experience GI distress. This stigma may restrain athletes from being truthful and giving a full and thorough insight into their experiences. Furthermore, there is a scarcity of similar research currently existing, which makes it challenging to compare findings and develop subsequent practical guidelines. This information would be particularly valuable to health professionals and practitioners to support them in effectively disseminating nutritional information to their target audience.

## 6 Conclusion

Over half of the participants of this study had previously experienced GISs at similar frequencies, regardless of whether during training or events. Sourcing nutrition information from qualified personnel was rarely employed by the participants. Comparably, most based their practices on previous experimentation and experiences. Both dietary and non-dietary strategies to manage GISs were scarce. Supplementing with nitrates or probiotics were the most frequently reported dietary strategies, while other and portion control were their non-dietary counterparts. Comparing these findings to previous research, recruiting the same cohort is a challenge due to the sparsity of such investigations. However, these findings are similar to those recruiting endurance athletes and further highlight the vast range of GISs such populations experience. In order to develop adequate dietary and non-dietary recommendations to support ultra-endurance athletes’ physiological adaptations while simultaneously alleviating GISs, it is crucial that the relevant professionals have an in-depth understanding of these GISs and the mechanisms behind them. As well as this, further research should investigate how best to communicate and disseminate such information to the ultra-endurance running community to support their implementation of evidence-based practices. This, in turn, will work to reduce individuals’ risks of developing long-term, chronic health complications resulting from poor GIS management and inadequate nutrition intake.

## Data availability statement

The raw data supporting the conclusions of this article will be made available by the authors, without undue reservation.

## Ethics statement

The studies involving humans were approved by ATU Research Subcommittee of Academic Council. The studies were conducted in accordance with the local legislation and institutional requirements. The participants provided their written informed consent to participate in this study.

## Author contributions

TR: Data curation, Formal analysis, Investigation, Methodology, Writing – original draft. ED: Formal analysis, Methodology, Supervision, Writing – review & editing. LR: Conceptualization, Formal analysis, Methodology, Supervision, Writing – review & editing.
