# Supplementary material for: Carbohydrate beliefs and practices of ultra-endurance runners in Ireland for gastrointestinal symptom management
Source: Front Nutr. 2024 May 31;11:1408101. doi: 10.3389/fnut.2024.1408101 (PMC11176419; doi:10.3389/fnut.2024.1408101)
Supplement: Supplementary file 1 [file Data_Sheet_1.pdf]

Lifestyle Habits of Ultra-marathon Runners in Ireland

Thank you for taking the time to complete this questionnaire. By completing this questionnaire, you will help us to better understand ultra-endurance athletes' sports nutrition beliefs, knowledge, information sources, and intended practices for training and events and how to prevent or manage gastrointestinal (gut) symptoms around exercise. If you are 18 years or over and you train or compete in events longer than 4 hours, you are invited to complete the questionnaire. The questionnaire will take about 20 minutes of your time. Please read the Research Participation Information Sheet (On next page, please READ) and confirm your consent to participate below.

PARTICIPANT INFORMATION SHEET

An invitation to participate

You are invited to partake in this study. Before you decide whether or not to participate, it is important for you to understand why this study is being conducted and what it will involve.

Please take the time to read the following information carefully. Thank you.

Title of Project (working title): An exploration of the lifestyle habits practiced by ultramarathon runners and recommended by their coaches.

Researcher Name & Email Address: Tansy Ryan – tansy.ryan@research.atu.ie or tansy.ryan@atu.ie

Head of Department Name: Dr Lisa Ryan – lisa.ryan@atu.ie

What is the purpose of the study?

The aim of the research is to develop a better understanding of the general everyday living of ultramarathon runners and lifestyle practices recommended by their coaches.

What would I be asked to do if I took part?

Participation will involve the undertaking of online questionnaires (approximately 30-40 mins duration) that will be sent to participants via email. Questions will be related to your general day-to-day living. If you are a coach, the final section will be asking about the lifestyle habits you recommend to your ultramarathon-running clients. Your responses may be included in the appendices of a research article, but your identity will be concealed, and responses will be completely anonymised.

Do I have to take part?

Participation in this study is voluntary. If you decide that you would like to take part, you will be asked to read and sign the attached consent form. You can decide, without any detriment to yourself, to withdraw your consent before your data is anonymised and amalgamated with the other data.

Will my information be kept confidential?

Your contact details and data will not be disclosed to anyone outside of this study. All data and any notes taken will be stored securely in a password-protected file saved onto an encrypted USB memory stick that is kept in a locked cabinet. All identifying data will be removed when the data is analysed and prior to the study publication. All personal data will be destroyed at the end of the examination process. All of the data is anonymous, and no personal or identifiable information is required as part of this study.

Next step – provide your consent if you are satisfied to do so

If you are happy to take part in this study after reading the information above, please complete the following.

1. Please read the Research Participation Information Sheet (Please READ) and confirm your consent to participate below.

- ◯ Yes, I have read it and I provide consent
- ◯ No, I do not provide consent

2. I have been given the opportunity to ask questions about the study.

- ◯ Yes
- ◯ No

3. I understand that my taking part is voluntary and that I can withdraw my consent at any time before my data is de-identified/amalgamated with other data.

- ◯ Yes
- ◯ No

4. I understand that, in any report on the results of this study, my identity shall remain anonymous.

- ◯ Yes
- ◯ No

5. What is your biological sex?

- ◯ Male
- ◯ Female
- ◯ Prefer not to disclose
- ◯ Other

6. What is your biological age?

- ◯ 18-24
- ◯ 25-34
- ◯ 35-44
- ◯ 45-54
- ◯ 54-66
- ◯ 66+

7. Please specify if the event you currently train for/compete in is a single or multi-day event?

- ◯ Single-day
- ◯ Multi-day

8. How long does it usually takes you to compete? Please give information in both hours and minutes.

[ ]

9. How would you best describe yourself and your level of training?

- ◯ Recreational, non-competitive
- ◯ Recreational, compete but not for placing
- ◯ Competitive at national level
- ◯ Competitive at an international level
- ◯ Compete at World events

10. During your heaviest training week, how many hours do you typically spend on aerobic endurance training for your sport or event?

- ◯ <10
- ◯ 10-20
- ◯ 20-30
- ◯ 30-50
- ◯ 50+

11. From the following list, select the food and drinks you recognise as carbohydrate or non-carbohydrate choices? (Please note: A response is required for each item)

	Carbohydrate choices	Non-carbohydrate choices
100g grilled chicken	◯	◯
2 slices of wholemeal bread	◯	◯
1 cup of raw rolled oats	◯	◯
1 medium baked potato	◯	◯
100g baked salmon	◯	◯
1 cup cooked pasta	◯	◯
1 cup cooked with rice	◯	◯
4 large leaves of iceberg lettuce	◯	◯
300mL of sports drink (eg Powerade)	◯	◯
1 large banana	◯	◯
300mL regular soft drink/ soda (eg coke or lemonade)	◯	◯
3 wheat biscuits (eg Weetabix)	◯	◯
1/2 large avocado	◯	◯
1 cup raw broccoli	◯	◯
2 boiled eggs	◯	◯

12. From the list below, please select the MOST important information source that has influenced your nutrition practices in relation to your sport.

- ◯ The internet - websites
- ◯ The internet - social media pages (of individuals/groups)
- ◯ Books
- ◯ Other athletes
- ◯ Coaches
- ◯ A qualified professional (ie sports nutritionist)
- ◯ Through my own experimentation
- ◯ Other

13. Select how much you agree or disagree with the following statements about endurance athletes consuming additional carbohydrates around training sessions or events to improve athletic performance.

Compared to a non-training day or rest day, endurance athletes would benefit from increasing their carbohydrate intake.......

	Strongly agree	Agree	Neutral	Disagree	Strongly disagree
1 – 2 days BEFORE an event 90 minutes (1.5 hours) or longer	◯	◯	◯	◯	◯
In the last meal or snack BEFORE endurance training or an event 90 minutes (1.5 hours) or longer	◯	◯	◯	◯	◯
DURING endurance training or an event (e.g. sports drinks or gels) lasting 60 minutes (1 hour) or longer	◯	◯	◯	◯	◯
Within 30 minutes AFTER endurance exercise during recovery when there are less than 8 hours between two fuel demanding sessions	◯	◯	◯	◯	◯
Statement 5	◯	◯	◯	◯	◯

14. Please consider what you plan to do for this scenario: **1-2 DAYS BEFORE AN EVENT 90 MINUTES OR LONGER** (in your main sport or event).

    Select from the drop-down boxes, whether you plan to eat LESS, the SAME, or MORE energy (calories/ kilojoules), carbohydrate, protein, or fat, compared to a non-training or lighter training day? N.B. This includes both solid and liquid foods.

	Plan to eat LESS	Plan to eat the SAME	Plan to eat MORE
Energy (calories/ kilojoules)	◯	◯	◯
Carbohydrates	◯	◯	◯
Protein	◯	◯	◯
Fat	◯	◯	◯

15. Please consider what you plan to do for this scenario: **IN THE LAST MEAL OR SNACK BEFORE ENDURANCE TRAINING OR AN EVENT 90 MINUTES OR LONGER** (in your main sport or event).

    Select from the drop-down boxes, whether you plan to eat LESS, the SAME, or MORE energy (calories/ kilojoules), carbohydrate, protein, or fat, compared to a non-training or lighter training day? N.B. This includes both solid and liquid foods.

	Plan to eat LESS	Plan to eat the SAME	Plan to eat MORE
Energy (calories/ kilojoules)	◯	◯	◯
Carbohydrates	◯	◯	◯
Protein	◯	◯	◯
Fat	◯	◯	◯

16. Please consider what you plan to do for this scenario: **DURING ENDURANCE TRAINING OR AN EVENT (ie sports gels and drinks) LASTING 60 MINUTES OR LONGER** (in your main sport or event).

    Select from the drop-down boxes, whether you plan to eat LESS, the SAME, or MORE energy (calories/ kilojoules), carbohydrate, protein, or fat, compared to a non-training or lighter training day? N.B. This includes both solid and liquid foods.

	Plan to eat LESS	Plan to eat the SAME	Plan to eat MORE
Energy (calories/ kilojoules)	◯	◯	◯
Carbohydrates	◯	◯	◯
Protein	◯	◯	◯
Fat	◯	◯	◯

17. Please consider what you plan to do for this scenario: **WITHIN 30 MINUTES AFTER ENDURANCE EXERCISE DURING RECOVERY WHEN THERE ARE LESS THAN 8 HOURS BETWEEN TWO FUEL DEMANDING SESSIONS** (in your main sport or event).

    Select from the drop-down boxes, whether you plan to eat LESS, the SAME, or MORE energy (calories/ kilojoules), carbohydrate, protein, or fat, compared to a non-training or lighter training day? N.B. This includes both solid and liquid foods.

	Plan to eat LESS	Plan to eat the SAME	Plan to eat MORE
Energy (calories/ kilojoules)	◯	◯	◯
Protein	◯	◯	◯
Carbohydrates	◯	◯	◯
Fat	◯	◯	◯

18. Please review the table and select all the dietary and non-dietary strategies you have tried to reduce your gastrointestinal symptoms.
NB: Please select "Not tried" if you have not tried that dietary strategy.
*FODMAPs (Fermentable Oligosaccharides Disaccharides, Monosaccharides, and Polyols)

	BEFORE exercise (Last meal or snack before exercise	DURING Exercise	AFTER exercise (Within 30 minutes of exercise completion	Dietary change made, but not specifically related to exercise	Dietary component SUCCESSFULLY reduced GIS	Dietary component DID NOT SUCCESSFULLY reduced GIS	Not tried to change this dietary component
Gluten-free	◯	◯	◯	◯	◯	◯	◯
Dairy-free	◯	◯	◯	◯	◯	◯	◯
Wheat-free	◯	◯	◯	◯	◯	◯	◯
Low FODMAPs	◯	◯	◯	◯	◯	◯	◯
Lactose-free	◯	◯	◯	◯	◯	◯	◯
Glutamine	◯	◯	◯	◯	◯	◯	◯
L-Citrulline	◯	◯	◯	◯	◯	◯	◯
Arginine	◯	◯	◯	◯	◯	◯	◯
Bovine Colostrum	◯	◯	◯	◯	◯	◯	◯
Curcumin	◯	◯	◯	◯	◯	◯	◯
Probiotics	◯	◯	◯	◯	◯	◯	◯
Prebiotics	◯	◯	◯	◯	◯	◯	◯
Symbiotics	◯	◯	◯	◯	◯	◯	◯
Antioxidants (Vitamin C or E)	◯	◯	◯	◯	◯	◯	◯
Nitrates (eg Beetroot juice)	◯	◯	◯	◯	◯	◯	◯

19. From the list below please select the severity of the different gastrointestinal symptoms you experience DURING training. (Note: NO response equals NO symptoms).
No symptoms = 0;
Mild symptoms = 1 – 4 (i.e. sensation of GIS, but not substantial enough to interfere with exercise workload);
Severe symptoms = 5 – 9 (i.e. GIS substantial enough to interfere with exercise workload);
Extremely severe symptoms = 10 (i.e. indicative of extreme GIS warranting exercise cessation).

Adapted from Modified Visual Analogue Scale, Gaskell, et al. (2019) IJSNEM.

	0 = No symptoms	1-4 = Mild symptoms	5-9 = Severe symptoms	10 = Extremely severe sympyoms
Belching	◯	◯	◯	◯
Defecation: diarrhoea	◯	◯	◯	◯
Flatulence	◯	◯	◯	◯
Projectile vomiting	◯	◯	◯	◯
Dizziness	◯	◯	◯	◯
Heartburn	◯	◯	◯	◯
Stomach pain	◯	◯	◯	◯
Left intestinal pain	◯	◯	◯	◯
Defecation: bloody stools	◯	◯	◯	◯
Regurgitation	◯	◯	◯	◯
Urge to defecate	◯	◯	◯	◯
Bloating (stomach fullness)	◯	◯	◯	◯
Nausea	◯	◯	◯	◯
Urge to regurgitate	◯	◯	◯	◯
Defecation: loose stools	◯	◯	◯	◯
Stitch (acute transient abdominal pain)	◯	◯	◯	◯
Lower abdominal bloating (abdominal pressure)	◯	◯	◯	◯

20. From the list below please select the severity of the different gastrointestinal symptoms you experience BEFORE competition. (Note: NO response equals NO symptoms).
No symptoms = 0;
Mild symptoms = 1 – 4 (i.e. sensation of GIS, but not substantial enough to interfere with exercise workload);
Severe symptoms = 5 – 9 (i.e. GIS substantial enough to interfere with exercise workload);
Extremely severe symptoms = 10 (i.e. indicative of extreme GIS warranting exercise cessation).

Adapted from Modified Visual Analogue Scale, Gaskell, et al. (2019) IJSNEM.

	0 = No symptoms	1-4 = Mild symptoms	5-9 = Severe symptoms	10 = Extremely severe sympyoms
Nausea	○	○	○	○
Regurgitation	○	○	○	○
Stomach pain	○	○	○	○
Projectile vomiting	○	○	○	○
Urge to defecate	○	○	○	○
Dizziness	○	○	○	○
Stitch (acute transient abdominal pain)	○	○	○	○
Defecation: diarrhoea	○	○	○	○
Bloating (stomach fullness)	○	○	○	○
Lower abdominal bloating (abdominal pressure)	○	○	○	○
Flatulence	○	○	○	○
Defecation: loose stools	○	○	○	○
Heartburn	○	○	○	○
Defecation: bloody stools	○	○	○	○
Urge to regurgitate	○	○	○	○
Left intestinal pain	○	○	○	○
Belching	○	○	○	○

21. With regards to the below stated dietary component, please select if you have tried eating more or less of to reduce the development of your gastrointestinal symptoms (GIS).
NB: Please select "Not tried" if you have not tried changing that dietary component.

FAT

	BEFORE exercise (Last meal or snack before exercise	DURING Exercise	AFTER exercise (Within 30 minutes of exercise completion	Dietary change made, but not specifically related to exercise	Dietary component SUCCESSFULLY reduced GIS	Dietary component DID NOT SUCCESSFULLY reduced GIS	Not tried to change this dietary component
Eat MORE							
Success of reducing GIS							
Eat LESS							
Eat the SAME							

22. With regards to the below stated dietary component, please select if you have tried eating more or less of to reduce the development of your gastrointestinal symptoms (GIS).
NB: Please select "Not tried" if you have not tried changing that dietary component.

CARBOHYDRATES

	BEFORE exercise (Last meal or snack before exercise	DURING Exercise	AFTER exercise (Within 30 minutes of exercise completion	Dietary change made, but not specifically related to exercise	Dietary component SUCCESSFULLY reduced GIS	Dietary component DID NOT SUCCESSFULLY reduced GIS	Not tried to change this dietary component
Eat the SAME							
Eat MORE							
Eat LESS							
Success of reducing GIS							

23. From the list below please select the severity of the different gastrointestinal symptoms you experience AFTER training. (Note: NO response equals NO symptoms).
No symptoms = 0;
Mild symptoms = 1 – 4 (i.e. sensation of GIS, but not substantial enough to interfere with exercise workload);
Severe symptoms = 5 – 9 (i.e. GIS substantial enough to interfere with exercise workload);
Extremely severe symptoms = 10 (i.e. indicative of extreme GIS warranting exercise cessation).

Adapted from Modified Visual Analogue Scale, Gaskell, et al. (2019) IJSNEM.

	0 = No symptoms	1-4 = Mild symptoms	5-9 = Severe symptoms	10 = Extremely severe sympyoms
Heartburn	○	○	○	○
Stitch (acute transient abdominal pain)	○	○	○	○
Flatulence	○	○	○	○
Left intestinal pain	○	○	○	○
Defecation: bloody stools	○	○	○	○
Urge to defecate	○	○	○	○
Dizziness	○	○	○	○
Urge to regurgitate	○	○	○	○
Nausea	○	○	○	○
Defecation: diarrhoea	○	○	○	○
Projectile vomiting	○	○	○	○
Bloating (stomach fullness)	○	○	○	○
Stomach pain	○	○	○	○
Lower abdominal bloating (abdominal pressure)	○	○	○	○
Defecation: loose stools	○	○	○	○
Belching	○	○	○	○
Regurgitation	○	○	○	○

24. From the list below please select the severity of the different gastrointestinal symptoms you experience DURING competition. (Note: NO response equals NO symptoms).
No symptoms = 0;
Mild symptoms = 1 – 4 (i.e. sensation of GIS, but not substantial enough to interfere with exercise workload);
Severe symptoms = 5 – 9 (i.e. GIS substantial enough to interfere with exercise workload);
Extremely severe symptoms = 10 (i.e. indicative of extreme GIS warranting exercise cessation).

Adapted from Modified Visual Analogue Scale, Gaskell, et al. (2019) IJSNEM.

	0 = No symptoms	1-4 = Mild symptoms	5-9 = Severe symptoms	10 = Extremely severe sympyoms
Stitch (acute transient abdominal pain)	○	○	○	○
Left intestinal pain	○	○	○	○
Defecation: diarrhoea	○	○	○	○
Defecation: loose stools	○	○	○	○
Urge to defecate	○	○	○	○
Urge to regurgitate	○	○	○	○
Dizziness	○	○	○	○
Bloating (stomach fullness)	○	○	○	○
Defecation: bloody stools	○	○	○	○
Belching	○	○	○	○
Heartburn	○	○	○	○
Regurgitation	○	○	○	○
Nausea	○	○	○	○
Stomach pain	○	○	○	○
Flatulence	○	○	○	○
Lower abdominal bloating (abdominal pressure)	○	○	○	○
Projectile vomiting	○	○	○	○

25. When do you experience gastrointestinal symptoms most frequently?

- ◯ Around (before, during and/or after) Training
- ◯ Around (before, during and/or after) Competitions
- ◯ Equally around training and competitions
- ◯ I do not experience them

26. From the list below please select the severity of the different gastrointestinal symptoms you experience AFTER competition. (Note: NO response equals NO symptoms).
No symptoms = 0;
Mild symptoms = 1 – 4 (i.e. sensation of GIS, but not substantial enough to interfere with exercise workload);
Severe symptoms = 5 – 9 (i.e. GIS substantial enough to interfere with exercise workload);
Extremely severe symptoms = 10 (i.e. indicative of extreme GIS warranting exercise cessation).

Adapted from Modified Visual Analogue Scale, Gaskell, et al. (2019) IJSNEM.

	0 = No symptoms	1-4 = Mild symptoms	5-9 = Severe symptoms	10 = Extremely severe sympyoms
Nausea	◯	◯	◯	◯
Projectile vomiting	◯	◯	◯	◯
Urge to defecate	◯	◯	◯	◯
Belching	◯	◯	◯	◯
Defecation: diarrhoea	◯	◯	◯	◯
Stitch (acute transient abdominal pain)	◯	◯	◯	◯
Urge to regurgitate	◯	◯	◯	◯
Bloating (stomach fullness)	◯	◯	◯	◯
Defecation: loose stools	◯	◯	◯	◯
Regurgitation	◯	◯	◯	◯
Lower abdominal bloating (abdominal pressure)	◯	◯	◯	◯
Left intestinal pain	◯	◯	◯	◯
Defecation: bloody stools	◯	◯	◯	◯
Stomach pain	◯	◯	◯	◯
Heartburn	◯	◯	◯	◯
Dizziness	◯	◯	◯	◯
Flatulence	◯	◯	◯	◯

27. From the list below please select the severity of the different gastrointestinal symptoms you experience BEFORE training. (Note: NO response equals NO symptoms).
No symptoms = 0;
Mild symptoms = 1 – 4 (i.e. sensation of GIS, but not substantial enough to interfere with exercise workload);
Severe symptoms = 5 – 9 (i.e. GIS substantial enough to interfere with exercise workload);
Extremely severe symptoms = 10 (i.e. indicative of extreme GIS warranting exercise cessation).

Adapted from Modified Visual Analogue Scale, Gaskell, et al. (2019) IJSNEM.

	0 = No symptoms	1-4 = Mild symptoms	5-9 = Severe symptoms	10 = Extremely severe sympyoms
Flatulence	○	○	○	○
Dizziness	○	○	○	○
Urge to regurgitate	○	○	○	○
Defecation: loose stools	○	○	○	○
Projectile vomiting	○	○	○	○
Left intestinal pain	○	○	○	○
Bloating (stomach fullness)	○	○	○	○
Defecation: diarrhoea	○	○	○	○
Nausea	○	○	○	○
Stomach pain	○	○	○	○
Lower abdominal bloating (abdominal pressure)	○	○	○	○
Belching	○	○	○	○
Stitch (acute transient abdominal pain)	○	○	○	○
Urge to defecate	○	○	○	○
Defecation: bloody stools	○	○	○	○
Regurgitation	○	○	○	○
Heartburn	○	○	○	○

28. With regards to the below stated dietary component, please select if you have tried eating more or less of to reduce the development of your gastrointestinal symptoms (GIS).
NB: Please select "Not tried" if you have not tried changing that dietary component.

PROTEIN

	BEFORE exercise (Last meal or snack before exercise	DURING Exercise	AFTER exercise (Within 30 minutes of exercise completion	Dietary change made, but not specifically related to exercise	Dietary component SUCCESSFULLY reduced GIS	Dietary component DID NOT SUCCESSFULLY reduced GIS	Not tried to change this dietary component
Eat MORE	○	○	○	○	○	○	○
Eat the SAME	○	○	○	○	○	○	○
Success of reducing GIS	○	○	○	○	○	○	○
Eat LESS	○	○	○	○	○	○	○

29. With regards to the below stated dietary component, please select if you have tried eating more or less of to reduce the development of your gastrointestinal symptoms (GIS).
NB: Please select "Not tried" if you have not tried changing that dietary component.

WATER OR FLUID

	BEFORE exercise (Last meal or snack before exercise	DURING Exercise	AFTER exercise (Within 30 minutes of exercise completion	Dietary change made, but not specifically related to exercise	Dietary component SUCCESSFULLY reduced GIS	Dietary component DID NOT SUCCESSFULLY reduced GIS	Not tried to change this dietary component
Eat the SAME	○	○	○	○	○	○	○
Eat MORE	○	○	○	○	○	○	○
Eat LESS	○	○	○	○	○	○	○
Success of reducing GIS	○	○	○	○	○	○	○

30. With regards to the below stated dietary component, please select if you have tried eating more or less of to reduce the development of your gastrointestinal symptoms (GIS).
NB: Please select "Not tried" if you have not tried changing that dietary component.

COFFEE OR CAFFEINE

	BEFORE exercise (Last meal or snack before exercise	DURING Exercise	AFTER exercise (Within 30 minutes of exercise completion	Dietary change made, but not specifically related to exercise	Dietary component SUCCESSFULLY reduced GIS	Dietary component DID NOT SUCCESSFULLY reduced GIS	Not tried to change this dietary component
Eat LESS	◯	◯	◯	◯	◯	◯	◯
Success of reducing GIS	◯	◯	◯	◯	◯	◯	◯
Eat MORE	◯	◯	◯	◯	◯	◯	◯
Eat the SAME	◯	◯	◯	◯	◯	◯	◯

31. With regards to the below stated dietary component, please select if you have tried eating more or less of to reduce the development of your gastrointestinal symptoms (GIS).
NB: Please select "Not tried" if you have not tried changing that dietary component.

FIBRE

	BEFORE exercise (Last meal or snack before exercise	DURING Exercise	AFTER exercise (Within 30 minutes of exercise completion	Dietary change made, but not specifically related to exercise	Dietary component SUCCESSFULLY reduced GIS	Dietary component DID NOT SUCCESSFULLY reduced GIS	Not tried to change this dietary component
Eat the SAME	◯	◯	◯	◯	◯	◯	◯
Success of reducing GIS	◯	◯	◯	◯	◯	◯	◯
Eat MORE	◯	◯	◯	◯	◯	◯	◯
Eat LESS	◯	◯	◯	◯	◯	◯	◯

32. Please select all of the other strategies you have tried to reduce your gastrointestinal symptoms, and whether you consider them successful or unsuccessful.

	Successful	Unsuccessful	Made symptoms worse	Have not tried
Medications	◯	◯	◯	◯
Relaxation/meditation	◯	◯	◯	◯
Herbal preparations	◯	◯	◯	◯
Acupuncture	◯	◯	◯	◯
Sports psychology	◯	◯	◯	◯
Portion size manipulation	◯	◯	◯	◯
All liquid diet pre-event	◯	◯	◯	◯
Other strategy	◯	◯	◯	◯

33. Do you suffer from a diagnosed gastrointestinal disease or disorder? If yes, please specify. If no, leave blank.

This content is neither created nor endorsed by Microsoft. The data you submit will be sent to the form owner.

Microsoft Forms
